# *Galleria mellonella* as a drug discovery model to study oxidative stress

**DOI:** 10.1038/s41598-025-99337-6

**Published:** 2025-04-30

**Authors:** Fred Jonathan Edzeamey, Zenouska Ramchunder, Ronan R. McCarthy, Sara Anjomani Virmouni

**Affiliations:** Department of Biosciences, College of Health, Medicine and Life Sciences, Brunel University of London, London, UK

**Keywords:** *Galleria mellonella*, Oxidative toxicity, Fibroblast cells, Antioxidants, In vivo, In vitro, Drug safety, Toxicology

## Abstract

Biological systems are equipped with endogenous antioxidant defence mechanisms against reactive oxygen species (ROS). Accumulation of ROS usually overwhelms this, creating pathologic effects. Oxidative toxicity has been reported as a causative factor in neurodegenerative diseases, cancer and diabetes mellitus (DM). However, developing an elaborate in vivo model system for mechanistic and therapeutic studies has been challenging. This present study sought to establish *Galleria mellonella* larvae as an ideal model for studying oxidative toxicity as a precursor to in vitro studies. We investigated Indole-3-propionic acid, Trolox, Resveratrol, Alpha tocopherol, Alpha lipoic acid, Orotic acid, Ginsenoside RB1, and Xanthohumol in this study, based on their antioxidant effects previously reported in different disease models. Tolerable concentrations of the compounds were established in vivo. Whilst no toxicity was recorded following treatment with Alpha tocopherol and Orotic acid, the remaining compounds displayed marked toxicity. We then conducted cell viability experiments in primary human fibroblast cell lines, and observed that tolerable concentrations in larvae produced 50–100% cell viability in vitro. Finally, Resveratrol and Alpha tocopherol were observed to rescue the larvae from juglone-induced oxidative toxicity. The larvae of *Galleria mellonella* can therefore be used for conducting oxidative toxicity and proof-of-concept studies of compounds.

## Introduction

Metabolic and cellular pathways in living organisms employ oxygen as their potent oxidising agent. Oxygen is reported as the most electronegative element with the strongest reducing capability^1^. However, the utilisation of oxygen is associated with the generation of reactive oxygen species (ROS). The generation of ROS and its accumulation in living organisms is controlled and neutralised by endogenous antioxidant defence systems. The effectiveness of these inherent antioxidant defence mechanisms is compromised by excessive generation and accumulation of ROS, which topples the endogenous defence mechanism^1^.

The pathological consequence of increased generation and accumulation of ROS is oxidative stress which plays paramount role in the pathogenesis of diseases like Alzheimer’s disease (AD), Parkinson’s disease (PD), Friedreich’s ataxia (FRDA), cancer and diabetes mellitus (DM), amongst others. Although this pathologic signature has been widely reported, the development of an elaborate system for in vivo mechanistic and therapeutic studies remains a challenge^2^. The discovery of the biomedical importance of the larvae of the greater wax moth, *Galleria mellonella* (*G. mellonella*) has led to further exploration of the larvae in replacing and augmenting previous model systems or used as a model for proof-of-concept studies to justify the progress of research into other tightly regulated model systems such as mammalian models^3,4^. Previously, the larvae were used to assess the acute toxicity of compounds which led to the identification of both toxic and non-toxic compounds^1^. Moya-Anderico et al. utilised the larvae to study the toxicity of nanoparticles^2^. The toxicity of 1-akyl-3-methylimidazolium chloride ionic liquids was also established using the larvae^3^. In addition, the larvae have been employed in determining the toxicity of okadaic acid^4^ and the toxicity and efficacy of new antimicrobial agents^5^. Furthermore, the larvae have been used to study the pathogenesis of several microbial diseases due to their susceptibility to human disease-causing pathogens^6^. The innate immunity of this insect model has been reported to have parts analogous to mammalian models with haemocytes as its immune cells^6^. These haemocytes can produce innate immune effects comparable to their mammalian counterparts^6^. This makes the model very useful in drug discovery and disease mechanism studies. The ease of procuring *G*. *mellonella* larvae, the relatively inexpensive cost, as well as the less demanding husbandry requirements, training, and equipment for their use establish *G. mellonella* as the most suitable insect model for this study when compared to other insect models^3,6^. Compounds selected for this present study are as follows: Indole-3-propionic acid (I3PA), Trolox (TX), Resveratrol (RESV), Alpha tocopherol (AT), Alpha lipoic acid (ALA), Orotic acid (OA), Ginsenoside RB1 (GnRB1) and Xanthohumol (XAN). I3PA is an antioxidant with no prooxidant properties which has been approved for the treatment of AD^7^. I3AP has been reported to exert its therapeutic effects by protecting neurons from oxidative damage occasioned by β-amyloid proteins^7^. I3PA has also been reported to mitigate oxidative stress due to ischemic damage in the hippocampus of ischemia-induced mice^8^. TX (6-hydroxy-2,5,7,8-tetramethylchroman-2-carboxylic acid) is a hydrophilic analogue of vitamin E possessing both antioxidant and prooxidant activities^9,10^. TX exerts its antioxidant activity via attenuating the polyunsaturated fatty acid peroxidation by binding and deactivating peroxyl radicals^9^. GnRB1 from Panax ginseng has been used in traditional medicine for the treatment of many diseases including microbial infections and inflammatory disorders^11^. GnRB1 has been reported to inhibit α-synuclein (α-syn) fibrillation; the main pathologic signature of PD^12^. RESV is a plant polyphenolic compound commonly found in grapes’ skin and seeds, red and white wine, and cocoa. This compound has been reported to have anti-inflammatory, antioxidant, anticancer and neuroprotective properties^13^. The antioxidant effects of RESV have been proposed to be via the upregulation of antioxidant enzymes and free-radical scavenging^13^. XAN is a flavonoid compound found in *Humulus lupulus*^14^. It has been used in Chinese traditional medicine for insomnia, pulmonary tuberculosis, and acute bacteria dysentery. XAN is reported to scavenge for ROS at relatively low concentrations whilst possessing pro-oxidative and pro-apoptotic properties at higher concentrations^15^. AT is the major natural antioxidant preventing the oxidation of biomembrane of cells via the prevention of peroxidation of polyunsaturated fatty acids^16^. ALA, a derivative of octanoic acid with antioxidant activity, functions as cofactor for mitochondrial enzymes and it is approved for the treatment of diseases such as diabetes and complications associated with cardiovascular diseases^17^. The antioxidant effect of ALA has been proposed to be via the scavenging of free radicals located in both hydrophobic and hydrophilic compartments. OA is a compound whose synthesis occurs de novo during the synthesis of pyrimidines in mammals^18^. In humans, the enzyme dihydroorotate dehydrogenase catalyses the synthesis of OA by converting dihydroorotate to Orotic acid. OA has been reported to enhance the metabolism of folic acid and vitamin B12, hence several studies have investigated its potential as an anti-anemic agent^19^.

Juglone (5-Hydroxy-1,4-naphthoquinone) (JUG) is a natural product synthesised from plants belonging to the Walnut family. In exploring its antimicrobial potential, Juglone has been applied in folk medicine in the treatment of varied diseases such as infections caused by viruses, bacteria, and fungi^20,21^. In *G. mellonella*, the feeding of the first instar with 0.5 to 3 mg of juglone mixed in 2 g of feed was reported to induce genotoxic and oxidative stress. This was evident by an increase in malondialdehyde level and decrease in endogenous antioxidant enzymes coupled with significant damage to DNA and chromosome extracted from the insect haemocytes^22,23^. The similarities between the innate immune systems of this insect model and mammals, make it an ideal biological model for testing toxicity and screening environmental toxicants^6^. Several toxicity studies have been conducted using the larvae. However, the utilisation of the larvae as a model to study oxidative toxicity has not been reported. The present study sought to establish the larvae of *G. mellonella* as an ideal model for studying oxidative toxicity. Moreover, we sought to establish a relationship between toxicity of the compounds in the larvae and human fibroblast cells.

## Materials and methods

### Compounds

Trolox (CAS:53188-07-1), Indole-3-propionic acid (CAS: 830-96-6), Orotic acid (CAS: 65-86-1), Alpha tocopherol (CAS: 10191-41-0), Alpha lipoic acid (CAS: 1077-28-7), Xanthohumol (CAS: 6754-58-1), Resveratrol (CAS: 501-36-0), Ginsenoside RB1 (CAS: 41753-43-9) and Juglone (CAS: 481-39-0) were all sourced from Sigma Aldrich. All compounds were solubilised in dimethyl sulfoxide (DMSO) except Alpha tocopherol which was solubilised in rapeseed oil.

### Cell lines

GM08399 and GM23976 were obtained from the Coriell Cell Repository (NJ, USA). H-Normal fibroblast cell line was a kind gift from Dr Terry Roberts (Brunel University London, London, UK). Details of the primary human fibroblast cell lines are summarised in Table 1.

**Table 1:** Details of human primary fibroblasts.

Cell line	Gender	Age (years)	Ethnicity
GM08399	Female	19	Caucasian
GM23976	Male	22	Caucasian
H-NORMAL	Female	20	Caucasian

### Cell culture

Cells were grown in Dulbecco’s Modified Eagle Medium (DMEM) 1X DMEM medium, supplemented with 10% fetal bovine serum (FBS) and 2% Pen-Strep (5000 U/ml penicillin and 5000 mg/ml of streptomycin, Fisher Scientific), at 37˚C, 5% CO_2_, before seeding at appropriate density for cell viability assay.

### In vivo multidrug toxicity studies in *G. mellonella*

The stock solution of each compound was prepared based on the solubility of the compound. Graded concentrations were then prepared from the stock concentration which represents the highest concentration down to the point where 100% survival was observed in the larvae. Larvae of *G. mellonella* were sourced from LiveFood UK Ltd. (Somerset, United Kingdom). The larvae were sorted onto an empty and sterile petri dish and were put into at least 4 groups (*n* = 10) per treatment. The Hamilton® syringe was disinfected in 70% ethanol and then washed twice with sterile PBS. 10 µL of each concentration of the compound [OA (50, 30, 25 and 15 mg/ml), RESV (50, 30, 25, 15, 10, 5, 7 mg/ml), I3PA (50, 30, 25 and 15 mg/ml), TX (50, 30, 25 and 15 mg/ml), XAN (3.5, 2.5, 1 and 0.5 mg/ml), GnRB1 (8, 7, 6 and 5 mg/ml), ALA (50, 25, 10 and 5 mg/ml), AT (500, 300, 200, 100, 50 and 25 mg/ml)] was injected into each larva in each group and the same volume of the vehicle was injected into the control group (*n* = 10). The larvae were incubated for 24-48 h at 37˚C. The number of dead larvae was counted within the first 24 h and at the end of the 48 h duration. Death was recorded when larva became black in colour (melanisation) and/or larva became immobile and unable to reorient when placed on the rear sides. For the induction of oxidative stress, larvae were injected concomitantly with 2 mg/ml (0.06 mg/g) Juglone and the compounds. Concentrations of the selected compounds were based on the results obtained from the toxicity studies. Mortality was then monitored at 6, 12, 18, 24, 36 and 48 h as previously described. To ensure blinding was maintained throughout the experiment, the drugs were coded so that the coding number became the unique identification code of the compound. The mortality was monitored and assessed by a researcher who was not present during the coding and injection of the larvae with the compound. The concentration of each compound in the larvae was reported as mg (mass of compound)/g (average weight of *G. mellonella*), calculated using the relation below.

$$\text{Concentration (mg/g) =} \, \frac{[\text{Mass of compound }\left({\text{mg}} \, \right) \, \text{/average weight of G. mellonella }\left({\text{g}}\right)\text{] }}{{10}{\text{0}}}.$$

The average weight of the larvae was 0.31 g. 10 µl of each compound was injected into the larvae and the concentration of each compound was in mg/ml.

### PrestoBlue cell viability assay

Human fibroblast cells were seeded into a 96 well plate (Corning) and incubated for 24 h. The medium was removed, and cells were washed with 1X PBS. Cells were incubated with the appropriate drug concentrations for 48 h. The cells were then washed with 1X PBS and pre-warmed medium containing 1X PrestoBlue Cell Viability Reagent (Invitrogen) was added followed by 3 h incubation at 37 °C, 5% CO_2_ and 95% humidity. The absorbance was then measured using a spectrophotometer (2000c, Invitrogen) at a wavelength of 570 nm with 600 nm reference. PrestoBlue cell viability reagent (Invitrogen) contains a non-fluorescent blue cell-permeant compound which is reduced by viable cells to a highly fluorescent red colour, which can be measured spectrophotometrically.

### Statistical analysis

Statistical tests were performed using GraphPad Prism (version 9). The unpaired two-tailed Student’s *t*-test was used to assess the significance of the differences between group data in the human fibroblasts, with a significance value set at *P* < 0.05, with data represented as mean ± SEM. For survival studies in the larvae, Kaplan–Meier curve was used, and percentage of survival was compared to control group using Log-rank (Mantel-Cox) test, with *P* < 0.05 considered significant. Data represent results from three independent experiments.

## Results

### Toxicity studies in *G. mellonella*

In this section, we present the tolerable dose, lethal dose (LD_50_) and the impact of doses below the LD_50_ for each compound in the larvae after injection, with mortality monitored over 48 h. The optimum doses observed were then used for subsequent experiments in the larvae.

**Fig. 1:**
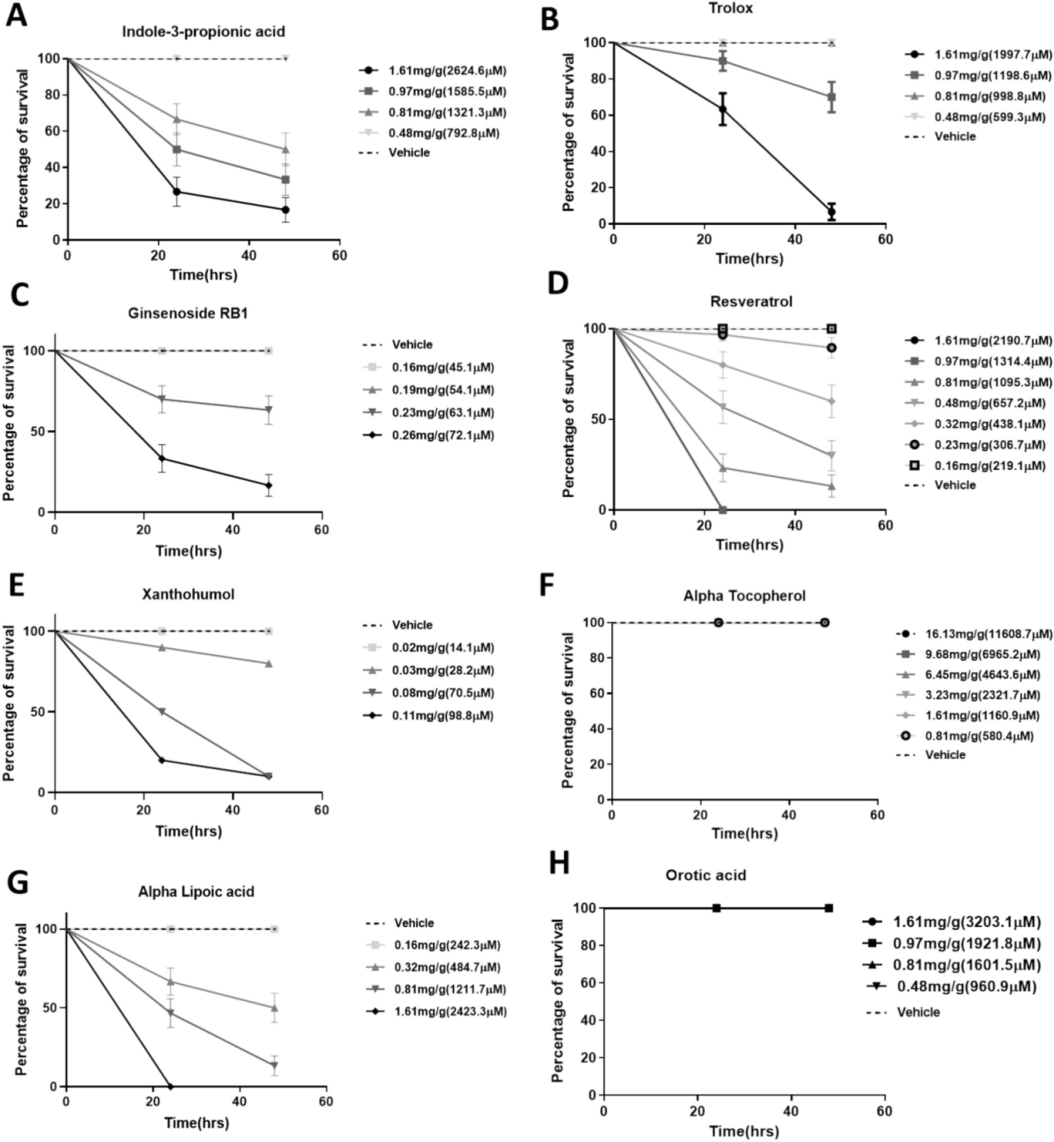
Toxicity of compounds in the larvae of *G. mellonella* within 48 h period. (**A**) Indole-3-propionic acid, (**B**) Trolox, (**C**) Ginsenoside RB1 (**D**) Resveratrol, (**E**) Xanthohumol, (**F**) Alpha tocopherol, (**G**) Alpha lipoic acid and (**H**) Orotic acid. Concentration represents mass of compound (mg) per average weight of *Galleria* (g). Data presented as percentage of survival, compared to control group using Log-rank (Mantel-Cox) test with *p* < 0.05 considered significant. Data represent results from three independent experiments, *n* = 30.

The percentage of survival (26.7%, *p* < 0.05) following the injection of 50 mg/ml (1.61 mg/g) I3PA at 24 h reduced to 16.7% at 48 h. 50% and 66.7% (*p* < 0.05) percentage survival were observed in larvae injected with 30 mg/ml (0.97 mg/g) and 25 mg/ml (0.81 mg/g) of the same compound within 24 h respectively. The survival of the larvae decreased to 33.3% and 50% at 48 h. The LD_50_ recorded for I3PA was 1.59 mg/g. No toxicity was observed in larvae injected with concentrations ≤ 15 mg/ml (0.48 mg/g) of this compound when compared to the vehicle (*p* > 0.999) (Fig. 1A), while concentrations below the LD_50_ but above 15 mg/ml (0.48 mg/g) were lethal.

Within 24 h following the injection of 50 mg/ml (1.61 mg/g) of TX, the percentage survival observed was 63% (*p* < 0.05) and this further reduced to 6.7% (*p* < 0.05) in 48 h. 90% percentage survival was observed in larvae injected with 30 mg/ml (0.97 mg/g) TX within 24 h period and this also declined to 70% in 48 h. However, no toxicity was recorded in the larvae injected with 25 mg/ml (0.81 mg/g) and 15 mg/ml (0.48 mg/g) (*p* > 0.999) within the entire 48 h period (Fig. 1B). 1.56 mg/g was recorded as the medial lethal dose with concentrations below this threshold but above 0.81 mg/g producing mortality in the larvae. Concentrations ≤ 0.81 mg/g produced a 100% survival in the larvae with no significant difference (*p* > 0.999) when compared to the vehicle.

The percentage of survival in larvae injected with 8 mg/ml (0.26 mg/g) Ginsenoside RB1 reduced from 33.3% within the first 24 h to 16.7% in 48 h (*p* < 0.05). Larvae injected with 7 mg/ml (0.23 mg/g) had 70% survival at 24 h which later decreased to 63.3% at 48 h period. The medial lethal dose observed was 0.24 mg/g. No toxicity was observed in larvae injected with 6 mg/ml (0.19 mg/g) and 5 mg/ml (0.16 mg/g) of the same compound (Fig. 1C). No significant difference (*p* > 0.999) was observed between the two lower concentrations and the vehicle.

The injection of larvae with 50 mg/ml (1.61 mg/g) and 30 mg/ml (0.97 mg/g) RESV produced 0% survival within 24 h with no significant difference between the two concentrations (*p* > 0.05). 25 mg/ml (0.81 mg/g) of the compound produced 23.3% survival within 24 h which decreased to 13.3% at the end of the 48 h duration. Within 24 h, 15 mg/ml (0.48 mg/g) and 10 mg/ml (0.32 mg/g) doses produced 56.7 and 80% survival which reduced to 30 and 60% respectively at the end of the 48 h period. There was no significant difference (*p* = 0.1241) between 25 mg/ml and 15 mg/ml, and between 15 mg/ml and 10 mg/ml doses (*p* = 0.3136). However, a significant difference (*p* = 0.0153) was observed between 25 mg/ml and 10 mg/ml RESV treatment groups. Whilst 7 mg/ml produced 96.7% survival within 24 h and 89.5% at 48 h, 5 mg/ml of the compound produced no toxicity in the larvae. No significant difference was observed between 7 mg/ml, 5 mg/ml and the vehicle groups (*p* > 0.05) (Fig. 1D). The LD_50_ recorded was 0.36 mg/g with concentration below the LD_50_ (0.36 mg/g to 0.24 mg/g) producing mortality in the larvae. The lethal concentrations of RESV in the larvae includes concentrations ≥ 0.24 mg/g.

16.7% survival was observed in larvae injected with 3.5 mg/ml (0.11 mg/g) XAN in 24 h and this was reduced to 3.3% at the end of the 48 h period, whilst 46.7% survival was observed in larvae injected with 2.5 mg/ml (0.08 mg/g) XAN which reduced to 10% in 48 h. There was a significant difference (*p* = 0.0138) between the 3.5 mg/ml and 2.5 mg/ml treatment groups. 1 mg/ml (0.03 mg/g) of the same compound produced an 80% survival in the larvae within 24 h and this was maintained at the end of the 48 h period. There was a significant difference between the 3.5 mg/ml and 1 mg/ml (*p* = 0.0010), and 2.5 mg/ml and 1 mg/ml (*p* = 0.0022) treatment groups. No toxicity was observed in larvae injected with 0.5 mg/ml (0.02 mg/g) XAN leading to no significant difference (*p* > 0.999) between the 0.5 mg/ml, 1 mg/ml, and the vehicle groups (Fig. 1E). The LD_50_ recorded was 0.05 mg/g and concentrations > 0.02 mg/g were observed as lethal.

The injection of different concentrations ranging from 25 mg/ml (0.81 mg/g) to 500 mg/ml (16.13 mg/g) of AT in the larvae of *G. mellonella* did not generate any toxic effects, producing a 100% survival in all treatment groups (Fig. 1F). There was no significant difference (*p* > 0.999) between the treatment and vehicle treated groups (*p* > 0.999). The injection of 50 mg/ml (1.61 mg/g) ALA into the *G. mellonella* larvae (*n* = 30) produced toxicity within 24 h with no survival. Larvae injected with 25 mg/ml (0.81 mg/g) of the same compound had 46.7% survival within the first 24 h and this reduced to 13.3% at the end of the 48 h duration. 66.7% survival was observed in larvae injected with 10 mg/ml (0.32 mg/g) of the compound within 24 h, however, the percentage survival reduced to 50% within 48 h. No toxicity was observed in larvae injected with 5 mg/ml (0.16 mg/g) ALA during the entire 48 h duration, thereby producing no significant difference (*p* > 0.999) between the 5 mg/ml ALA treatment and vehicle groups (Fig. 1G). The LD_50_ of 0.35 mg/g was recorded for ALA treatment in the larvae with mortality observed with concentrations > 0.16 mg/g.

The percentage of survival observed following the injection of the larvae with 50 mg/ml (1.61 mg/g) to 15 mg/ml (0.48 mg/g) of OA was 100% (*p* > 0.999), indicating no toxic effects of the drug (Fig. 1H). There was no significant difference between the drugs and vehicle treated groups.

### Compounds with no rescue effect on Juglone induced-oxidative toxicity in *G. mellonella* larvae

**Fig. 2:**
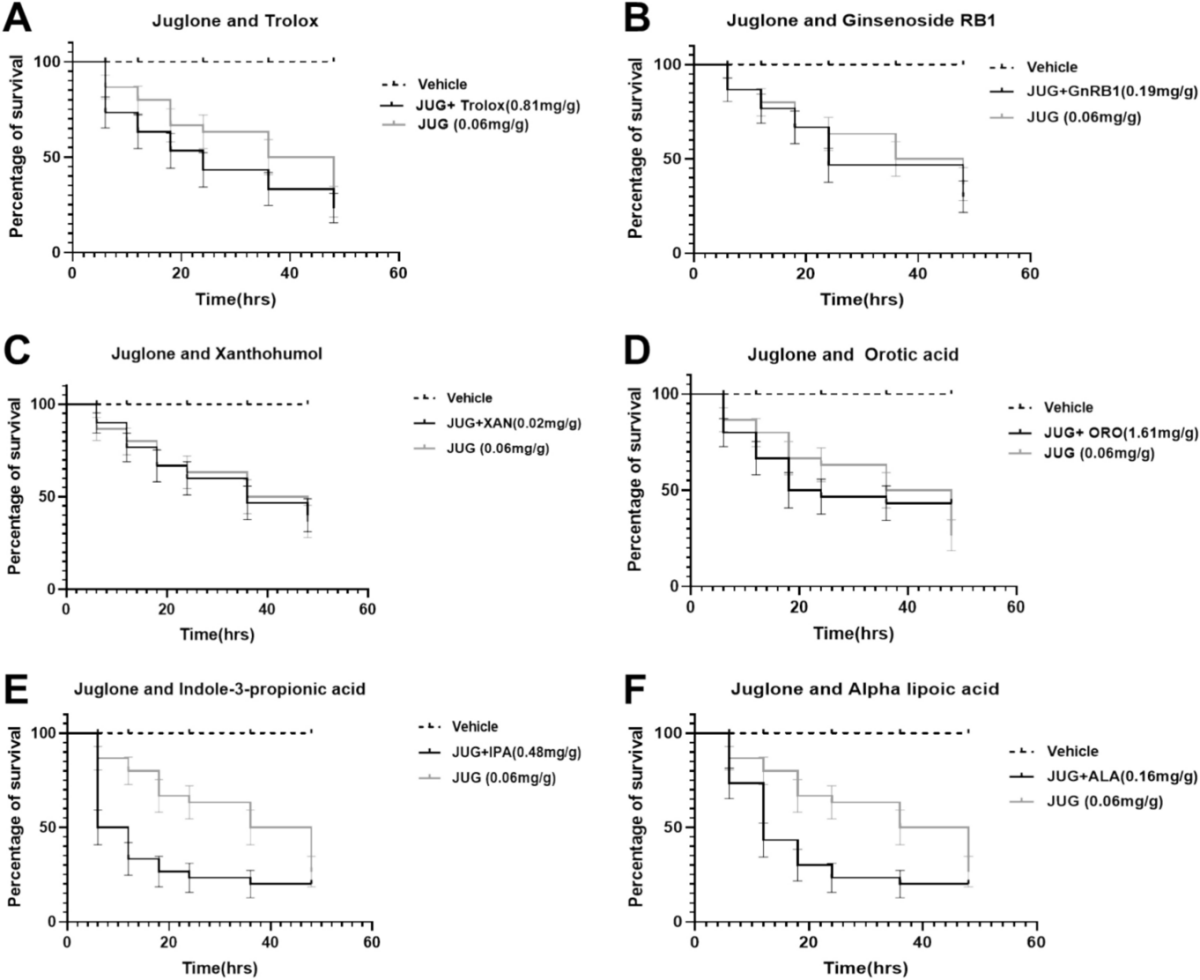
Effects of compounds on Juglone induced-oxidative toxicity in *G. mellonella* larvae within 48 h period. (**A**) Trolox (TX), (**B**) Ginsenoside RB1 (GnRB1), (**C**) Xanthohumol (XAN), (**D**) Orotic acid (OA), (**E**) Indole-3-propionic acid (I3PA) and (**F**) Alpha lipoic acid (ALA). Concentration represents mass of individual compound (mg) per average weight of Galleria (g). Data presented as percentage of survival, compared to vehicle group using Log-rank (Mantel-Cox) test with *p* < 0.05 considered significant. Data represent results from three independent experiments, *n* = 30.

After establishing the tolerable doses of the compounds in the larvae, we further investigated the ability of the compounds to rescue the larvae from mortality due to oxidative toxicity. We injected the larvae with Juglone (JUG), followed by the injection with the tolerable dose of each compound. Larvae injected with JUG + TX (Fig. 2A), JUG + GnRB1 (Fig. 2B), JUG + XAN (Fig. 2C), and JUG + OA (Fig. 2D) were shown no rescue effects on the oxidative toxicity induced by larvae. In addition, there were no significant differences (*p* > 0.05) between the JUG alone and JUG + TX, JUG + GnRB1, JUG + XAN, and JUG + OA treatment groups. However, a significant reduction in percentage survival was recorded in larvae injected with JUG + I3PA (Fig. 2E) and JUG + ALA (Fig. 2F). I3PA and ALA were therefore observed to further increase mortality in the larvae in the presence of JUG.

### RESV and AT rescues larvae from Juglone induced-oxidative toxicity

**Fig. 3:**
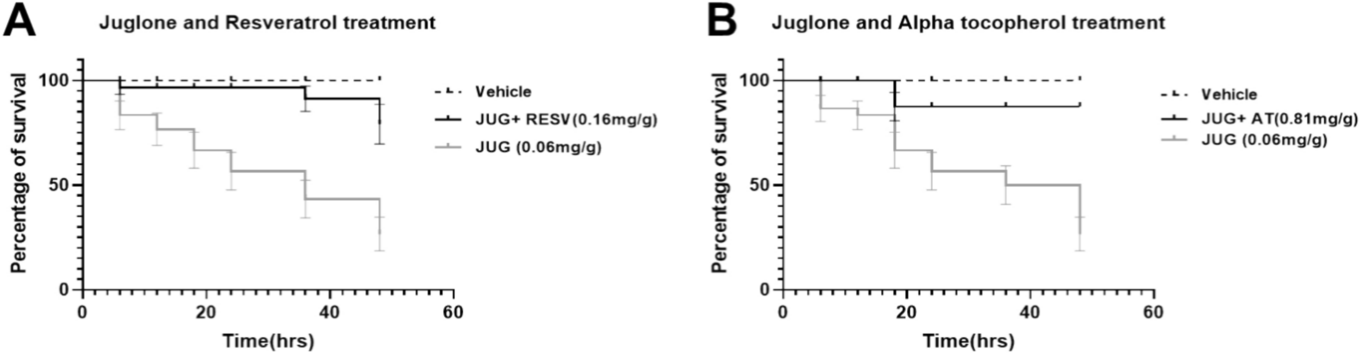
Mortality rescue effects of compounds following Juglone-mediate oxidative toxicity in the larvae of *G. mellonella* within 48 h period. (**A**) Resveratrol (RESV), (**B**) Alpha tocopherol (AT), Concentration represents mass of individual compound (mg) per average weight of Galleria (g). Data presented as percentage of survival, compared to vehicle group using Log-rank (Mantel-Cox) test with *p* < 0.05 considered significant. Data represent results from three independent experiments, *n* = 30.

The treatment of larvae with both 0.06 mg/g Juglone and 0.16 mg/g RESV was observed to maintain a 100% survival of larvae until 36 h where a 91.2 percentage survival was recorded (Fig. 3A). This was further reduced to 79.1% at the end of the 48 h period. A statistical significance effect (*p* = 0.0004) was observed between the JUG alone and JUG + RESV treated groups. The difference in survival between the JUG + RESV treated and vehicle groups was observed to be statistically significant (*p* = 0.0147). 0.81 mg/g AT, when injected after the 0.06 mg/g JUG, was observed to prevent mortality up to 18 h where 87.5 percentage survival was recorded. The 87.5% survival of the larvae was maintained until the end of the 48 h period. While a statistical difference (*p* = 0.001) was observed between JUG alone and JUG + AT treated groups, no significant difference was observed between the JUG + AT and vehicle treated groups (*p* = 0.0563) (Fig. 3B).

### PrestoBlue cell viability studies in human fibroblast cells

Cell viability studies were conducted in human fibroblast cells with the same compounds used in the insect larvae. We also included concentrations which were tolerable in the larvae to evaluate their effects in the fibroblast cells. This was to establish the tolerable concentration of the compounds in the human fibroblast cells. We sought to compare and establish a relationship between toxicity of the compounds in human fibroblast cells and the larvae of *G*. *mellonella*.

**Fig. 4:**
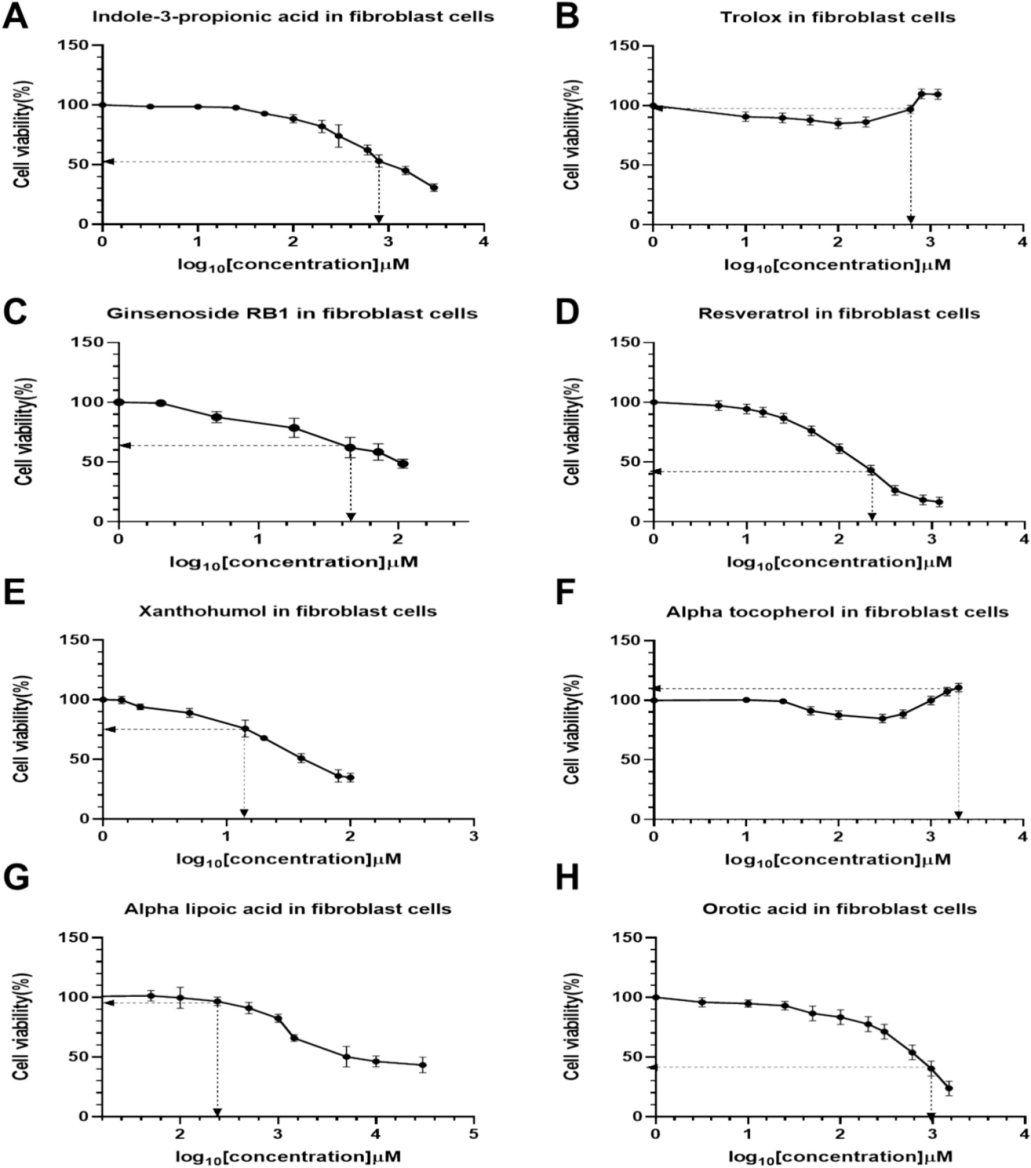
Cell viability studies of human fibroblasts after 48 h treatment with compounds using PrestoBlue Cell Viability Reagent. (**A**) Indole-3-propionic acid (I3PA), (**B**) Trolox (TX), (**C**) Ginsenoside RB1 (GnRB1), (**D**) Resveratrol (RESV), (**E**) Xanthohumol (XAN), (**F**) Alpha tocopherol (AT), (**G**) Alpha lipoic acid (ALA) and (**H**) Orotic acid (OA). Three human fibroblast cell lines from healthy individuals were used. The mean value of all data set was normalised to the PrestoBlue reduction of the vehicle (pegged at 100%). Error bars indicate SEM, and all data are presented as mean ± SEM from three independent experiments. The X-axis indicates the log_10_ of the various concentrations. The dotted lines represent the tolerable concentrations of the compounds in the larvae of *G. mellonella*. Significant difference between vehicle and treated groups were analysed using students’ *t*-test, *p* < 0.05 was considered to be statistically significant.

No significant reduction (*p* > 0.05) in cellular viability was recorded in fibroblast cells treated with 10 μM to 300 μM I3PA. However, there was a significant reduction (*p* < 0.05) in fibroblast cells treated with 600 µM-1.2 mM of I3PA (Fig. 4A). Cells treated with TX were observed to have tolerated 10–100 µM concentration as there was no significant impact on their viabilities (*p* > 0.05). Although an increase in cell viability was observed in cells treated with 400 μM to 1.2 mM TX, this was not statistically significant (*p* > 0.05) (Fig. 4B). 15 μM to 108 μM of GnRB1 was observed to significantly reduce cell viability (*p* < 0.05) whilst the cells tolerated concentrations less than 10 µM GnRB1 (Fig. 4C). Cells treated with RESV from 50 μM to 1.2 mM experienced a significant reduction (*p* < 0.05) in cellular viability whilst no significant reduction in cellular viability was observed in cells treated with 5 μM to 25 μM (Fig. 4D). The cells were shown a significant decrease in their cellular viability when treated with 10–130 µM XAN, whilst 0.1-10 μM doses had no significant effects on cellular viability (Fig. 4E). While no significant reduction (*p* > 0.05) in cell viability was observed in cells treated with AT (10–500 μM), treatment of cells with 1 mM-2 mM AT led to an increase in cell viability (Fig. 4F). The cell lines were sensitive to concentrations greater than 1000 µM, thereby significantly reducing their cellular viability whist no significant effect on cellular viability was observed in cells treated with concentrations of ALA less than 1000 µM (Fig. 4G). 50 μM-1.5 mM OA significantly reduced the viability of cells whilst the cells tolerated 25–30 μM OA, with no significant reduction in cellular viability (Fig. 4H).

### Toxicity of compounds in larvae versus toxicity in human fibroblast cells

As reported earlier, we conducted toxicity studies in both the larvae and human fibroblast cells with the same compounds. In the human fibroblast cells, we included the concentrations which were recorded to have been tolerated by the larvae; thus, the minimum concentrations which produced a 100% viability in the larvae. The concentration of TX (599.3 µM) and ALA (242.3 µM) which produced a 100% survival in the larvae was observed to produce a 96.9% viability in the human fibroblast cell lines. Table 2 shows the toxicity of the compounds in the larvae and human fibroblast cells.

**Table 2:** Comparison on the toxic effects of the same concentration of the compounds in the insect larvae of *G. mellonella* and human fibroblast cell lines.

Compound	Concentration/µM	% Survival in larvae	% Cell viability in human fibroblast cells
Resveratrol	219.1	100	44.8
Indole-3-propionic acid	798.2	100	52.9
Trolox	599.3	100	96.9
Alpha tocopherol	580.4	100	110
Alpha lipoic acid	242.3	100	96.9
Ginsenoside RB1	45.1	100	64.6
Orotic acid	960.9	100	40.2
Xanthohumol	14.1	100	74.9

Results represent the treatment of the larvae and cell lines for 48 h.

## Discussion

The ability of the larvae to tolerate these compounds at different doses was tested and the outcomes did not differ from what is expected of a biological system^3^. Whilst the larvae were able to tolerate some doses of the compounds, notably ≤ 0.48 mg/g of I3PA (Fig. 1A), ≤ 0.81 mg/g of Trolox (Fig. 1B), ≤ 0.19 mg/g of GnRB1 (Fig. 1C), ≤ 0.02 mg/g of XAN (Fig. 1E.), and ≤ 0.16 mg/g of RESV and ALA (Fig. 1D and G respectively), toxicity in the larvae was also recorded following treatments above the aforementioned doses. Some of the compounds (AT and OA) (Fig. 1F and H respectively) were found not to establish any toxic effect in the larvae even at their highest doses. The biological systems in mammals have been reported to metabolise compounds (usually mediated by the cytochrome P450 enzymes), thereby releasing metabolites or toxins which could result in the breakdown of various cellular components, thus leading to cell death^24–27^. Although some similarities have been drawn between the mammalian system and that of *G. mellonella*^1^, it has also been reported that the larvae may not metabolise some of the compounds hence no toxicity is exhibited by the larvae^1^. Other researchers have also opined that metabolism of compounds might have taken place, but the metabolites produced may have no toxic effects on the subcellular structures in the larvae^1,28^. The toxicities recorded in the larvae could be associated with two factors: the larvae responding to stress from the injection and the build-up of the compounds at those high doses^3^. However, the stress response due to the needle injection could be rule out since the vehicle group recorded no mortality. In this circumstance, the toxicities recorded in the larvae could be associated with the build of high doses of the compounds^3^. It is reported that the accumulation of the compounds could generate genotoxic stress leading to the destruction of subcellular components in the larvae^29,30^. The destruction of these cellular components might have been the trigger of mortality in the larvae.

In investigating the ability of the compounds to rescue the larvae from oxidative mortality, Juglone, a compound known to induce oxidative and genotoxic stress in Galleria^23,31^, was used to identify protective effects of the compounds. Injection of Juglone was followed by the injection with the tolerable doses of the compounds. Whilst some of the compounds (RESV and AT) (Fig. 3A and B respectively) were able to rescue the larvae from mortality occasioned by juglone, the injection of TX (Fig. 2A), GnRB1 (Fig. 2B), XAN (Fig. 2C), OA (Fig. 2D), I3PA (Fig. 2E) and ALA (Fig. 2F) exacerbated the toxicity in the larvae, with I3PA (Fig. 2E, p < 0.05) and ALA (Fig. 2F, p < 0.05) producing mortality with a statistical difference between the juglone alone and juglone + antioxidant groups. For compounds which rescued the larvae from oxidative mortality, it could be proposed that the improvement in survival was due to the activation of antioxidant response system in the larvae for instance; the protection of cellular membrane against lipid peroxidation in the case of alpha tocopherol^32–34^, and the upregulation of ARE-regulated genes and radical-scavenging in the case of resveratrol^35,36^ as these compounds have been reported to have antioxidant activity. In this study we show that the larvae can also be used for conducting proof-of-concept studies to establish the effect of compounds before proceeding into other complex and tightly regulated model systems. By comparing the toxicity of the compounds in the larvae and human fibroblast cell lines, we observed that the larvae could be used to determine the optimum concentrations of ALA and TX in human fibroblast cells since the tolerable concentrations in the larvae was quite similar to that in the fibroblast cells. However, for compounds like RESV, I3PA, and OA, it was observed that the tolerable concentration in the larvae almost reduced the cell viability in the fibroblast cells by 50%. We also observed that concentration of AT which has no toxic effect on the larvae could also be well tolerated in the fibroblast cell lines.

**Fig. 5:**
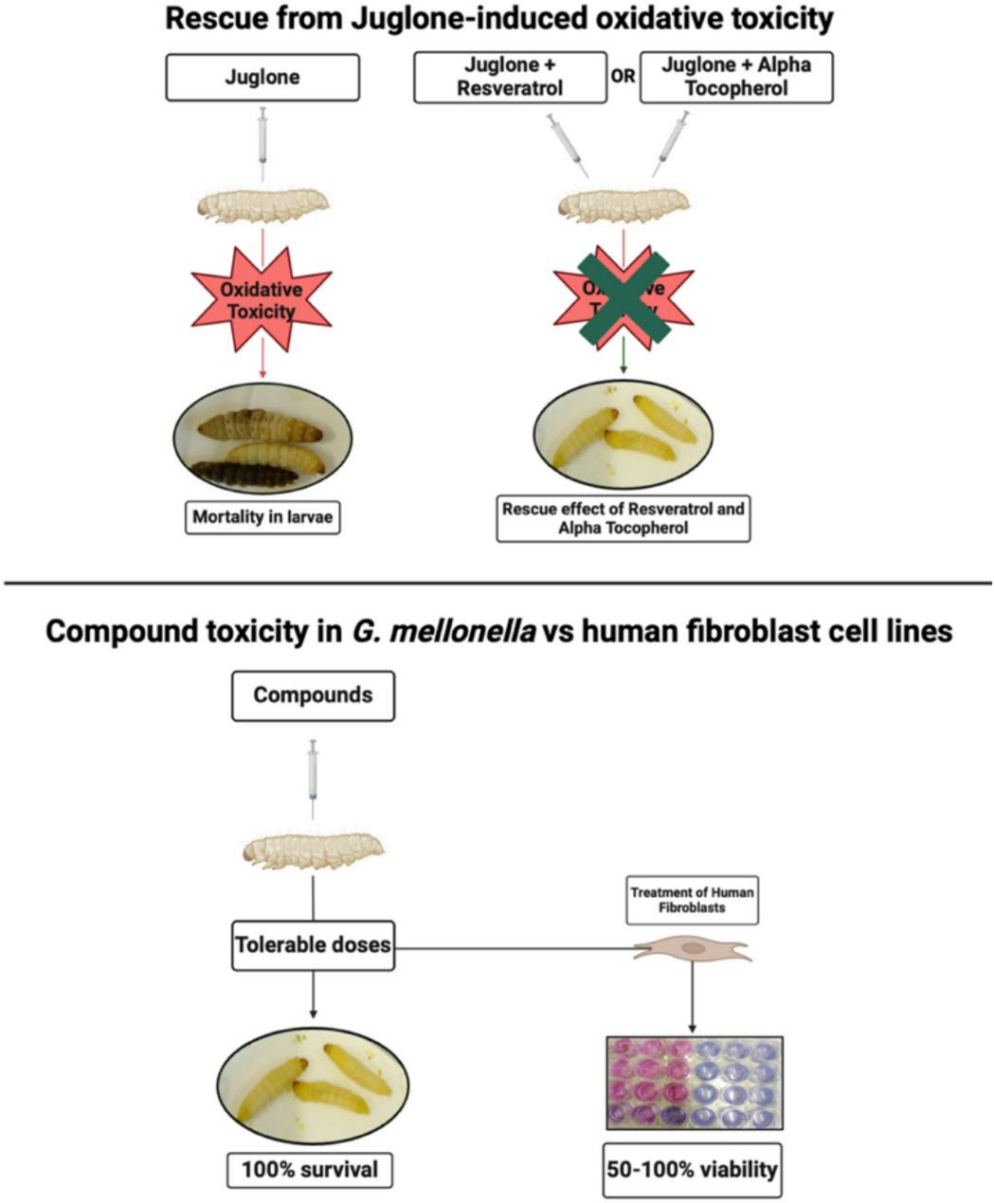
Summary of the rescue effect of RESV and AT in *G. mellonella* and their toxicity profile in human fibroblast cell lines. Treatment with RESV or AT significantly reduced juglone-induced mortality in *G. mellonella*. The maximum tolerable doses identified in vivo were subsequently used for in vitro studies with human fibroblast cell lines. At these doses, cell viability ranged from 50 to 100%. This image was created with BioRender. (https://BioRender.com).

In this study, we have observed that mortality rates of larvae treated with juglone + RESV or juglone + AT were lower than those larvae treated with just juglone. In addition, six of the antioxidants tested in the larvae exhibited 50% or greater cell viability when tested in the human fibroblast cell lines, with three of these showing cell viability greater than 95%. These findings are significant as they identify a possibility of using concentrations of antioxidants tested in *G. mellonella* to optimise safe and tolerable concentrations for human fibroblast cell lines (Fig. 5). However, one limitation of our study is that we have not yet identified the mechanism by which antioxidants, Particularly RESV and AT, improve the survival rate of larvae. Since juglone was used to induce oxidative stress, and led to increased mortality rate, it can be suggested that the oxidative stress may have contributed to the decreased survival rate, and that the antioxidant properties of RESV and AT helped to improved survival. Future studies could focus on elucidating the mechanisms by which RESV and AT exert their protective effects *G. mellonella* survival. Previous studies have used haemolymph of *G. mellonella* to identify oxidative stress biomarkers and measure antioxidant enzyme activity^37,38^, and this method could be employed to further explore how RESV and AT affect oxidative stress. Nevertheless, the findings of this study suggest that *G. mellonella* can be a valuable model for investigating the safety and tolerability of antioxidant, with the potential to translate these concentrations into in vitro models to streamline the optimisation process. To our knowledge, this is the first study to explore the use of *G. mellonella* to identify safe and tolerable concentrations for in vitro testing, and further investigation could help develop this model for optimising dosing in more complex in vivo models.

Altogether, the larvae could be used to predict the optimum-dosing regimen in human fibroblast cell lines and other cell lines with similar characteristics. Since it is cheaper working with the larvae as compared to other model systems, determining the LD_50_ and optimum concentrations of compounds could be done in the larvae as this will give the researchers a predictive range of concentrations to use for in vitro and in vivo toxicity studies. This could help reduce cost and time involved in establishing the tolerable concentration of compounds.

## Data Availability

The datasets used and/or analysed during the current study available from the corresponding author on reasonable request.
